# The effect of high oral loading dose of cholecalciferol in non-alcoholic fatty liver disease patients. A randomized placebo controlled trial

**DOI:** 10.3389/fphar.2023.1149967

**Published:** 2023-03-14

**Authors:** Amal Ahmed Mohamed, Ahmed Abdel Halim, Sahar Mohamed, Seham Mohamed Mahmoud, Eman Mohamed Bahgat Eldemiry, Rasha Sobh Mohamed, Mahmoud Maamoun Shaheen, Gina G. Naguib, Nashwa M. Muharram, Mona G. Khalil, Salma Saed, Randa Ibrahim, Ahmed Salah Seif, Noha Kamal, Karima Nasraldin, Ali Elsaid Abdelrahman, Radwa El Borolossy

**Affiliations:** ^1^ Department of Biochemistry and Molecular Biology, National Hepatology and Tropical Medicine Research Institute, Cairo, Egypt; ^2^ Tropical Medicine Department, National Hepatology and Tropical Medicine Research Institute, Cairo, Egypt; ^3^ Tropical Medicine Department, El Sahel Teaching Hospital, Cairo, Egypt; ^4^ Fellow of Clinical Pharmacology, Faculty of Pharmacy, Cairo University Hospitals, Cairo, Egypt; ^5^ Internal Medicine Department, Faculty of Medicine, Cairo University, Cairo, Egypt; ^6^ Department of Internal Medicine, Faculty of Medicine, Ain Shams University, Cairo, Egypt; ^7^ Medical Biochemistry and Molecular Biology, Faculty of Medicine, Menoufia University, Menoufia, Egypt; ^8^ Pharmacology and Toxicology Department, Faculty of Pharmacy, Modern University for Technology and Information, Cairo, Egypt; ^9^ Clinical and Chemical Pathology Department, Faculty of Medicine, Cairo University, Cairo, Egypt; ^10^ Clinical and Chemical Pathology Department, Nutrition Institute, Cairo, Egypt; ^11^ Tropical Medicine Hepatology and Gastroenterology Department, Shebeen El-Kom Teaching Hospital, Menoufia, Egypt; ^12^ Clinical Pathology Department, Theodor Bilharz Research Institute (TBRI), Ministry of Scientific Research and Higher Education, Gulf Medical University (GMU), Cairo, Egypt; ^13^ Faculty of Biotechnology, Modern Science and Arts University, Cairo, Egypt; ^14^ Diagnostic and Intervention Radiology, National Hepatology and Tropical Medicine Research Institute, Cairo, Egypt; ^15^ Department of Clinical Pharmacy, Faculty of Pharmacy Ain Shams University, Cairo, Egypt

**Keywords:** non-alcoholic fatty liver, Cholecalciferol, Steatosis, lipid profile, hsCRP

## Abstract

**Background and Aim:** Non-alcoholic fatty liver (NAFLD) is one of the most common progressive metabolic disorders worldwide. There are increasing scientific interests nowadays for the association between vitamin D status and Non-alcoholic fatty liver. Earlier studies have revealed that vitamin D deficiency is highly prevalent in Non-alcoholic fatty liver patients that contributes to poor outcomes. Hence, the present study aimed to assess the efficacy and safety of oral cholecalciferol on Non-alcoholic fatty liver patients.

**Subjects and Methods:** This study was conducted on 140 patients that were randomized either to group 1 that received the standard conventional therapy in addition to placebo or group 2 that received the standard conventional therapy in addition to cholecalciferol during the 4 months study period.

**Results:** At the end of the study group 2 revealed significant decrease (*p* < 0.05) in the mean serum level of TG, LDL-C, TC, hsCRP as compared to their baseline results and group 1 results. Additionally, a significant improvement in the serum levels of ALT (*p* = 0.001) was seen in group 2 at the end of the study when compared to group 1. Whereas group 1 did not show any change in these parameters when compared to group 2 and their baseline results.

**Conclusion:** Cholecalciferol was shown to have beneficial effects on serum ALT levels, hsCRP levels and lipid profile of NAFLD patients.

**Clinical Trial Registration:** https://prsinfo.clinicaltrials.gov/prs-users-guide.html, identifier NCT05613192

## 1 Introduction

Non-alcoholic fatty liver disease (NAFLD) is a metabolic disorder with high prevalence in patients suffering from chronic liver diseases (Chalasani et al., 2012). NAFLD is defined as the presence of more than 5% of fat deposits in the hepatocytes (hepatic steatosis) with no known other reasons of steatosis as high alcohol intake (Benedict and Zuhang, 2017).

The global prevalence of NAFLD differ according to the population reaching 13% in Africa, 32% in middle East and 30% in the United States (Ofosu et al., 2018). More than 70% of patients with metabolic syndrome suffer from NAFLD due to the excessive fat accumulation in this syndrome (Tolman et al., 2007). This disease usually begins as hepatic steatosis that may progress to steatohepatitis (NASH) with hepatic cells inflammation and may finally end with Chronic liver disease with fibrosis and cirrhosis (Agrawal and Duseja, 2014).

The main aetiology of NAFLD is associated with interaction between different factors as: environmental, genetic, hormonal and nutritional factors. Obesity and metabolic syndrome (MS) are considered the most common risk factors for NAFLD initiation, also they are linked to greater progression of the disease (Younossi et al., 2018). As visceral obesity constitutes a major health problem, it is now important for hepatologists to weigh risk factors that lead to insulin resistance and hepatic steatosis (Finelli and Tarantino, 2012). Visceral obesity and its adipose-tissue-resident macrophages produce many inflammatory cytokines that induce insulin resistance and play a great role in hepatic steatosis and fibrosis pathogenesis (Larter and Farrell, 2006).

Till now, the standard treatment for NAFLD is weigh reduction with life style modification (European Association for the Study of the Liver EASLEuropean Association for the Study of Diabetes EASDEuropean Association for the Study of Obesity EASO, 2016; Liyanagedera et al., 2017). However, there is no pharmacological management has been approved yet by guidelines. Accordingly, several therapies with different modes of action for treatment of NAFLD are gaining significant interests and are currently under clinical evaluation (Del Ben et al., 2014). Various pharmacological approaches using existing drugs have also been considered in the management of NAFLD and NASH. These attempts mainly focus on antidiabetics, anti-obesity drugs, antioxidants, and cytoprotective agents, including insulin sensitizers (e.g., metformin), thiazolidinediones (e.g., pioglitazone), glucagon-like peptide-1 (GLP-1) receptor agonists (e.g., liraglutide), a natural dihydroxy bile acid (e.g., ursodeoxycholic acid) or antioxidants (vitamin E) (Harrison et al., 2020; Negi et al., 2022).

One of these interesting therapies is cholecalciferol (native vitamin D) which is a fat-soluble vitamin that is endogenously produced in the skin, it exerts many beneficial effects other than its primary role in bone homeostasis (Barchetta et al., 2017). Vitamin D has been demonstrated by many animal and clinical studies to induce anti-inflammatory and anti-fibro genic activity in the liver through inhibiting proinflammatory cytokines, profibrotic mediators and oxidative stress (Abramovitch et al., 2011; Ding et al., 2013; Abramovitch et al., 2015; Beilfuss et al., 2015). Moreover, Vitamin D has been shown in several experimental studies to be an effective modulator of insulin sensitivity and metabolism of free fatty acids (FFAs). Hence, vitamin D deficiency (VDD) increase the percentage of FFAs circulating in the blood stream which promote fat deposition into the hepatocytes causing NAFLD (Barchetta et al., 2011).

Several clinical studies have proved the association between low vitamin D levels and NAFLD, also it correlates with the spectrum of inflammation and fibrosis that occur in the course of NAFLD (Targher et al., 2007; Manco et al., 2010; Nobili et al., 2014; Zhai et al., 2016).

VDD is defined as a serum 25-hydroxyvitamin D levels ≤20 ng/mL and it is very common in adults over 20 years. It can be attributed to many factors as: poor sunlight exposure, insufficient intake of food containing vitamin and malabsorption syndromes (Matthias and Micheal, 2013).

Patients with NAFLD have 26% additional risk to VDD as compared to controls owing to the impairment of 25 (OH)D synthesis due to the presence of steatosis (Eliades et al., 2013), in addition vitamin D receptor (VDR) expression in the hepatocytes decreases as the extent of the disease increase (Barchetta et al., 2012).

Sunlight therapy and vitamin D have shown clinical benefit in experimental animal models with fatty liver. Hence vitamin D supplementation can represent a simple and cheap therapy for the management of NAFLD (Geier, 2011).

In 2016, the first pilot prospective clinical trial was conducted to assess the effect of 24 weeks high-dose (25,000 IU/Week) oral cholecalciferol supplementation on the liver histological findings of 12 non-cirrhotic NASH patients, no beneficial effects of this treatment were found on the laboratory parameters of hepatic damage and insulin sensitivity (Kitson et al., 2016). After this study, other clinical trials were conducted evaluating the effect of oral Vitamin D with different dosing regimens in NAFLD, however results from these clinical studies are debatable (Sharifi et al., 2014; Barchetta et al., 2016; Lorvand Amiri et al., 2017).

Hence, our study aimed to determine the effect and safety of high oral loading dose of cholecalciferol supplementation on the clinical parameters related to liver steatosis, glycaemic control, insulin resistance and metabolic profile in NAFLD patients. According to our knowledge this is the first randomized placebo-controlled trial to investigate the impact of high oral loading dose of vitamin D in NAFLD patients.

## 2 Patients and methods

### 2.1 Study design

The present study was prospective, simply randomized (*via* computer generated sequence), placebo controlled double blinded study (patients, physicians, radiologist remained blinded from randomization) conducted on NAFLD patients in the outpatient liver clinics of the National Hepatology and Tropical Medicine Research institute, Cairo, Egypt. From March 2022 to August 2022.

### 2.2 Study population

Patients had to fulfil the following inclusion criteria to be included in the study: either male or female adult patients (>19 years) with fatty liver diagnosis by using upper abdominal ultrasound echography (US) and with T2D diagnosed according to ADA 2022 criteria (American Diabetes Association, 2022) and treated with metformin.

The main exclusion criteria from the study were as follows: Pregnant and/or lactating women, excessive alcohol use (as defined by an average daily consumption of alcohol >30 g/day in men and >20 g/day women), patients with other causes of chronic liver disease as viral hepatitis, drug induced hepatitis, autoimmune hepatitis, patients suffering of chronic kidney disease, hyper/hypoparathyroidism, hypersensitivity to cholecalciferol, hypercalcemia, patients taking supplementation with vitamin D, calcium and medications affecting calcium/vitamin D metabolism (as: anticonvulsants, glucocorticoids, antacids).

### 2.3 Study intervention

**FIGURE 1:**
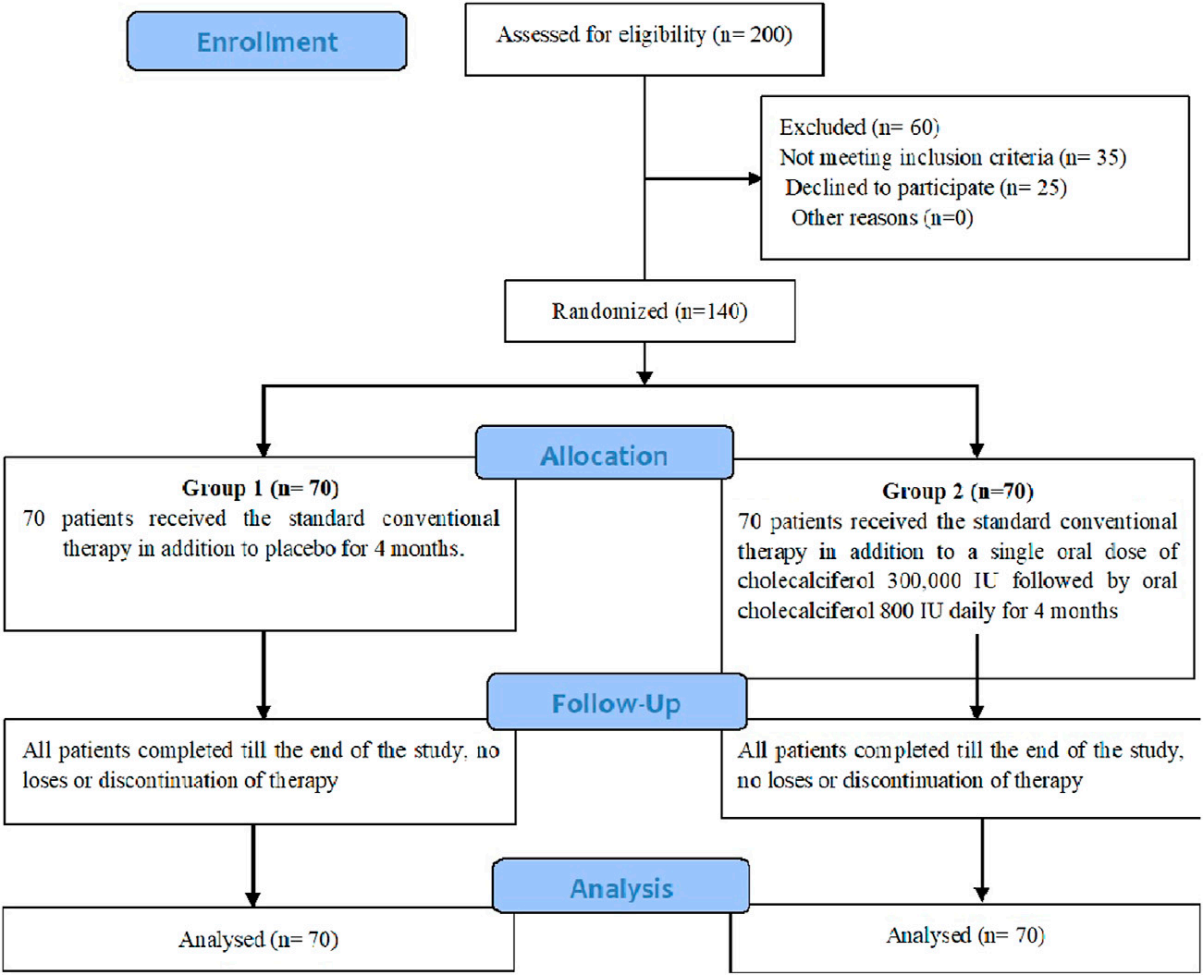
CONSORT flow diagram of patient allocation.

Hundred and forty eligible patients were included into the study and randomized by simple randomization into either of the two groups (Figure 1):

Group 1: 70 patients received the standard conventional therapy in addition to placebo for 4 months.

Group 2: 70 patients received the standard conventional therapy in addition to a single oral dose of cholecalciferol 200,000 IU (Devarol- S^®^, manufactured by Memphis company, Egypt) followed by oral cholecalciferol 800 IU (Vidrop^®^, manufactured by Medical Union Pharmaceuticals company, Egypt) daily for 4 months.

The standard conventional therapy in both groups included regular exercise in the form of any physical activity as: walking, cycling, *etc.*, For 30–45 min at least 5 days per week in addition to calorie restriction in overweight and obese patients (1,000–1,200 kcal/day for women, 1,200–1,500 kcal/day for men).

Study medications were given to the patients by an unblinded pharmacist to ensure the right treatment assignment, however this pharmacist was not included in the outcome assessment.

All patients were diagnosed with NAFLD depending on abdominal ultrasonography performed by a radiologist where the liver brightness and liver parenchyma with diffuse echogenicity in confirm the diagnosis.

### 2.4 Study procedures

The following information was collected from the patients including: age, sex, history of smoking or alcohol use, medications use, Sun exposure and vitamin D containing food consumption.

The grade of fatty liver (Kim et al., 2019) was classified as none (0), mild (1), moderate (2), or severe (3) according to the findings of liver brightness, hepatorenal echo contrast, deep attenuation of the ultrasound signal, and the blurring of vessels. The grading was recorded two times; the first at the beginning of the study and the second time after 4 months of cholecalciferol administration.

### 2.5 Laboratory measurements and clinical assessments

All patients were subjected to anthropometric measurements at baseline and at the end of the study including: height (meters), weight (kilograms), waist circumference (WC) (measured midway between the 12th rib and the iliac crest in inches). Body mass index (BMI) (measured as weight in kg divided by height in m^2^). According to WHO criteria: overweight is defined as BMI ≥25 kg/m^2^, obesity is defined as BMI ≥30 kg/m^2^ (Garvey et al., 2016).

Moreover, 10 mL Blood samples were collected from all patients at the beginning and at the end of the study after an overnight fasting, then blood samples were centrifuged at 3,000 rpm for sera separation for 10 min, and then sera were kept frozen at −80°C for analysis.

The following laboratory tests were measured

a- Glycaemic control: Fasting blood glucose (FBG mg/dl), glycated haemoglobin (HbA1C%), Fasting insulin (mU/L), Insulin resistance index calculated by the homeostasis model assessment insulin resistance (HOMA-IR) method using the product of fasting insulin and fasting plasma glucose divided by 405. The cut off value of HOMA-IR is more than 1.64.
b- Liver function tests: Alanine transaminase (ALT U/L), Aspartate transaminase (AST U/L), Albumin (g/dL), Gamma glutamyl transferase (GGT U/L), Alkaline phosphatase (ALP U/L).
c- Lipid Profile: Low density lipoprotein (LDL-C mg/dl), High density lipoprotein (HDL-C mg/dl), Triglycerides (TG mg/dL), Total cholesterol (TC mg/dL).
d- Other markers: High sensitivity C reactive protein (hsCRP mg/dl), Alfa fetoprotein (AFP ng/ml), serum 25-hydroxy vitamin D (25(OH) D ng/ml).

Analysis of FBG, ALT, AST, GGT, ALP, HDL-C, TG, TC was performed by enzymatic colorimetric methods, while analysis of serum 25(OH) D, fasting insulin, hsCRP and AFP was performed by enzyme linked immunosorbent assay technique (ELISA) (EIA-5240; DRG International Inc., Springfield., United States). Patients were considered to be vitamin D deficient when the level of 25(OH)D is less than 20 ng/mL, insufficient when the level is less than 21–29 ng/mL and when the level is 30 ng/mL or more, patients were considered to be sufficient or normal.

All Patients were followed up every 2 weeks by the clinical pharmacist in charge through patient encounter to ensure the compliance to the treatment regimen and to assess any adverse side effects.

### 2.6 Outcomes

#### 2.6.1 Primary outcomes

The improvement in the glycaemic control parameters, liver function tests, lipid profile and serum 25-hydroxy vitamin D of the patients at the end of the study

#### 2.6.2 Secondary outcomes

The Decrease in degree of steatosis on US with the improvement in CRP, AFP at the end of the 4 months.

### 2.7 Ethical consideration

The study protocol was revised and approved for the scientific and ethical issues by the institution review board of the ethical committee of National Hepatology and Tropical Medicine Research institute, Cairo, Egypt (serial number:10–22). The study was registered in Clinical trial. Gov (Identifier: NCT05613192). The study procedures were carried out in accordance with Good Clinical Practice guidelines, and the ethical principals in 2013 Helsinki Declaration. This study also applied CONSORT guidelines and ICMJE recommendations. All patients included in the study were informed and educated about the study protocol before their participation and requested to sign a written informed consent without any obligation to withdraw if they want to.

### 2.8 Statistical analysis

Sample size calculation was done depending on data from a previous study (Barchetta et al., 2016) by considering serum LDL-C as a key dependent variable, type I error of 0.05, and study power of 90%. Based on the suggested formula for parallel clinical trials, we reached the sample size of 50 patients in each group. Taking into account a possible drop-out rate of 30%, 70 patients will be enrolled in each group.

Statistical analysis was performed using the SPSS statistical program (v.22; SPSS, Chicago, IL). Mean and standard deviation (SD) were used to express the parametric data, and the categorical data were expressed as numbers and percentage. Data analysis were performed by the Paired Student’s *t*-test, Unpaired Student’s *t*-test and Chi-square test. The probability of error of 0.05 was considered to be significant, and 0.001 to be highly significant.

## 3 Results

### 3.1 Patients characteristics

**FIGURE 2:**
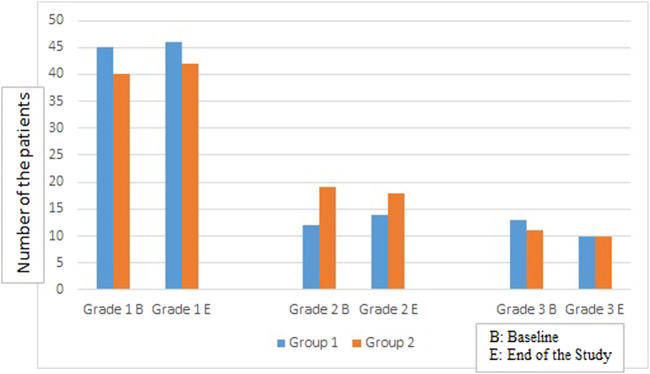
Comparison of the number of patients in both groups in different liver steatosis grades at baseline and at the end of the study.

All the 140 patients who started the study, completed till the end and there were no dropouts (Figure 1). At baseline no significant differences were found between both groups regarding the demographic data, anthropometric measures, laboratory measurements and degree of liver steatosis on US (Table 1; Figure 2). Totally 112 out of 140 patients had mean serum 25(OH) D < 30 ng/mL, in group 1 30 (43%) patients were vitamin D deficient and 25 (36%) were vitamin D insufficient while in group 2 40 (57%) patients were vitamin D deficient and 17 (24%) were vitamin D insufficient. Concerning vitamin D containing food consumption (fish beef liver. etc.), all patients in both groups stated that they have little amount due to financial burden.

**TABLE 1:** Comparison of demographic data, anthropometric measures and laboratory measurements in the two studied groups at baseline.

	Group 1 (*n* = 70)	Group 2 (*n* = 70)	*p*-Value
Age (years) Gender	54 ± 9.8	52 ± 10	0.09
Male *n* (%)			
Female *n* (%)	25 (36%)	29 (41%)	0.065
Weight (kg)	45 (64%)	41 (59%)	
BMI (kg/m^2^)	80.3 ± 4.6	85.5 ± 6.9	0.12
WC (cm)	31.3 ± 5.2	32.1 ± 7.8	0.32
Male	120 ± 5.3	119 ± 5.2	0.45
Female	118 ± 4.7	116 ± 5.2	0.39
FBG (mg/dL)	114 ± 9.9	120 ± 11.3	0.065
Fasting insulin (mU/L)	10.3 ± 3.9	9.5 ± 2.9	0.21
HOMA-IR	2.9 ± 1.7	2.8 ± 1.2	0.52
TC (mg/dL)	188 ± 22.5	179 ± 20.1	0.083
TG (mg/dL)	170 ± 30.2	175 ± 34	0.82
LDL-C (mg/dL)	150 ± 24	155 ± 65	0.07
HDL-C (mg/dL)	46 ± 4.9	45 ± 5.9	0.16
Male	45 ± 5.1	44 ± 6.9	0.15
Female	58 ± 9.1	61 ± 11.2	0.53
AST (U/L)	65 ± 8.2	66 ± 10	0.91
ALT (U/L)	46 ± 3.8	44 ± 4.9	0.72
ALP (U/L)	54 ± 5.9	60 ± 3.7	0.069
GGT (U/L)	3.9 ± 0.5	3.8 ± 0.32	0.13
Albumin (g/dL)	17 ± 1.4	15 ± 1.23	0.25
25(OH) D (ng/mL)	16 ± 1.2	16 ± 1.5	0.3
Male	18 ± 2.1	15 ± 1.3	0.4
Female	15.5 ± 1.6	15 ± 1.1	0.5
Age (19–30)	17 ± 1.8	17 ± 1.6	0.3
Age (30–60) hsCRP (mg/dL)	10.2 ± 0.4	11 ± 0.9	0.41
AFP (ng/mL)	15 ± 1.7	14 ± 1.9	0.09

Data are represented as mean ± SD or *n* (%). BMI, body mass index; WC, waist circumference; FBG, fasting blood glucose; HOMA-IR, homeostasis model assessment insulin resistance; TC, total cholesterol; TG, triglycerides; LDL-C, low density lipoprotein cholesterol; HDL-C, high density lipoprotein; AST, aspartate transaminase; ALT, alanine transaminase; AL, alkaline phosphatase; GGT, gamma glutamyl transpeptidase; hsCRP, High sensitive C- reactive protein; AFP, alfa fetoprotein. *p-value ≤ 0.05.

### 3.2 Laboratory measurements and clinical assessments

After 4 months of cholecalciferol supplementation, group 2 revealed significant decline in the mean serum level of TG (152 ± 25 vs. 175 ± 34), LDL-C (135 ± 15 vs. 155 ± 65), TC (150 ± 17.2 vs. 175 ± 20.1) as compared to their baseline results and group 1 results (Table 2). In addition, hsCRP mean serum level showed significant decrease in group 2 compared to the baseline results (7.9 ± 0.899 vs. 11 ± 0.9) and compared to group 1 (Table2). Moreover, a highly significant improvement in the serum levels of ALT (45 ± 5.1 vs. 66 ± 10) was seen in group 2 at the end of the study when compared to group 1 (Table 2). Whereas group 1 did not show any change in these parameters at the end of the study when compared to their baseline results.

**TABLE 2:** Comparison of anthropometric measures and laboratory measurements in the two studied groups at end of the study.

	Group 1 (*n* = 70)	Group 2 (*n* = 70)	*p*-Value
Weight (kg)	75 ± 11	80 ± 9.5	0.078
BMI (kg/m^2^)	30.3 ± 4	30.8 ± 5.2	0.92
WC (inches)	110 ± 3.9	112 ± 4.8	0.76
Male	111 ± 4.1	111 ± 3.8	0.91
Female	115 ± 11	119 ± 8.9	0.092
FBG (mg/dL)	10 ± 4	9 ± 2	0.76
Fasting insulin (mU/L)	2.8 ± 0.3	2.6 ± 0.1	0.85
HOMA-IR	180 ± 20.1	150 ± 17.2	0.03*
TC (mg/dL)	166 ± 24.2	152 ± 25	0.05*
TG (mg/dL)	149 ± 20	135 ± 15	0.043*
LDL-C (mg/dL)	43 ± 2.6	47 ± 10	0.06
HDL-C (mg/dL)	54 ± 7.9	59 ± 8.2	0.078
AST (U/L)	59 ± 6.2	45 ± 5.1	0.001**
ALT (U/L)	47 ± 4	45 ± 3.7	0.51
ALP (U/L)	55 ± 3.9	56 ± 2.1	0.36
GGT (U/L)	4 ± 0.6	4 ± 0.4	0.9
Albumin (g/dL)	19 ± 2.1	25 ± 3.1	0.05*
25(OH) D (ng/mL)	18 ± 2.4	24 ± 2.9	0.05*
Male	19 ± 1.9	25 ± 3.1	0.05*
Female	17 ± 1.87	24.5 ± 2.6	0.05*
Age (19–30)	18 ± 2.1	26 ± 2.4	0.05*
Age (30–60) hsCRP (mg/dL)	9.9 ± 0.8	7.9 ± 0.899	0.001*
AFP (ng/mL)	16 ± 2.4	14.5 ± 2.2	0.09

Data are represented as mean ± SD or *n* (%). BMI, body mass index; WC, waist circumference; FBG, fasting blood glucose; HOMA-IR, homeostasis model assessment insulin resistance; TC, total cholesterol; TG, triglycerides; LDL-C, low density lipoprotein cholesterol; HDL-C, high density lipoprotein; AST, aspartate transaminase; ALT, alanine transaminase; ALP, alkaline phosphatase; GGT, gamma glutamyl transpeptidase; hsCRP, High sensitive C- reactive protein; AFP, Alfa fetoprotein. *p-value ≤ 0.05, **p-value ≤ 0.001.

There was no change observed in the auxological parameters, BMI, glycemic control markers, AFP, AST, ALP, GGT in both groups at the end of the study (Table 2), also no significant difference was reported regarding the degree of liver steatosis on US in both groups (Figure 2).

Regarding serum 25(OH) group 2 reported significant increase as compared to their baseline results (25 ± 3.1 vs. 15 ± 1.23) and when compared to group 1 (19 ± 2.1 vs. 17 ± 1.4) after 4 months of cholecalciferol administration. 50 patients in group 2 became Vitamin D sufficient, 20 patients were found to be vitamin D insufficient and no patient was found in vitamin D deficient category at the end of the study. However, there were no change observed in group1 as compared to their baseline values.

Neither of the patients in group 2 reported any side effects after 4 months of daily cholecalciferol administration.

## 4 Discussion

NAFLD is one of the most common and prevalent progressive metabolic disorder worldwide (Charlton et al., 2011). It is manifested in different clinical spectrum that can start with simple fatty liver and ends with cirrhosis (Wong et al., 2015). Meanwhile, there are increasing scientific interests in the association between NAFLD and vitamin D levels (Pacifico et al., 2019). Vitamin D is a fat-soluble vitamin and an important component in many tissues and metabolic process with several functions extending beyond the skeletal homeostasis (Liu et al., 2019). Findings of earlier studies have revealed that vitamin D deficiency is highly prevalent in NAFLD patients and this contributes to poor outcomes and progression to liver cirrhosis (Targher et al., 2007; Manco et al., 2010; Nobili et al., 2014; Zhai et al., 2016). Also, several clinical trials evaluated the impact of cholecalciferol in NAFLD patients but till now there is no clear evidence on its beneficial effect in NAFLD patients (Sharifi et al., 2014; Barchetta et al., 2016; Kitson et al., 2016; Lorvand Amiri et al., 2017). Different vitamin D dosing regimens were investigated in these trials but none of these trials evaluated the impact of high oral loading dose followed by daily dose supplementation although the high oral vitamin D dosing was demonstrated to be superior to the daily regimen in treating hypovitaminosis in patients with different inflammatory diseases (Sainaghi et al., 2013; Wong et al., 2015; Mak et al., 2016). Hence our aim in the current study was to evaluate the impact of high oral loading dose vitamin D supplementation in NAFLD patients. The present study showed that vitamin D dosing significantly (*p* < 0.05) decreased serum ALT at the end of the study but there were no changes seen in other liver enzymes biomarkers and this was also shown in Sakpal et al. (2017) were the serum level of ALT decreased (*p* < 0.001) from 87 ± 48 to 59 ± 32 IU/mL after 6 months of vitamin D supplementation and in Lorvand Amiri et al. (2017) where ALT significantly decreased at the end of the study, whereas other studies reported no significant effects on ALT (Sharifi et al., 2014; Barchetta et al., 2016; Foroughi et al., 2016; Kitson et al., 2016). The discrepancy between our study results and other studies maybe due to the differences in the study population and the dosing regimen. Moreover, our study showed beneficial effect of vitamin D on the lipid profile including significant reduction in serum TG, LDL-C, TC. In accordance, previously Lorvand Amiri et al. (2017) and Sharifi et al. (2014) reported the reduced effect of vitamin D on TG, TC, LDL-C serum levels. On the other hand, other studies (Barchetta et al., 2016; Foroughi et al., 2016; Kitson et al., 2016) failed to show significant effect of vitamin D on the lipid profile. In addition, we reported significant decrease in the serum level of hsCRP from 11 ± 0.9 to 7.9 ± 0.89 mg/dL which was also reported in Foroughi et al. (2016), Sharifi et al. (2014) In contrast Barchetta et al. (2016) and Sakpal et al. (2017) did not show significant effect on CRP after daily vitamin D. Furthermore, concerning the glycemic index and anthropometric parameters, our trial did not reveal any beneficial effect of vitamin D on all these parameters at the end of the study. Likewise, all previous trials (Sharifi et al., 2014; Barchetta et al., 2016; Foroughi et al., 2016; Kitson et al., 2016; Mak et al., 2016; Lorvand Amiri et al., 2017) did not report any significant changes in the anthropometric measures. Conversely, two older studies (Foroughi et al., 2016; Lorvand Amiri et al., 2017) revealed a significant reduction in FBG, HOMA-IR at the end of vitamin D supplementation duration.

Further, no change in the degree of liver steatosis on US was found in both groups at the end of the study, similarly Barchetta et al. (2016) did not report significant difference in the hepatic fat fraction measured by magnetic resonance after 24 weeks of oral high-dose vitamin D supplementation in T2D patients with NAFLD.

Hypovitaminosis D was found in 80% of our NAFLD patients and this was supported by earlier trials (Targher et al., 2007; Manco et al., 2010; Nobili et al., 2014; Zhai et al., 2016) that demonstrated the association between low serum 25(OH) and NAFLD.

There are two limitations in our study; the lack of liver biopsy due to financial constraints so we cannot follow the histological changes in the liver and the short duration of the study. Further studies with longer duration evaluating the histological changes that occur in the liver with vitamin D supplementation are required.

## 5 Conclusion

In conclusion, our randomized placebo-controlled trial demonstrated that hypovitaminosis D is common in NAFLD patient and high oral loading dose followed by daily oral doses of vitamin D had beneficial effects on serum ALT levels, hsCRP levels and lipid profile of NAFLD patients.

## Data Availability

The raw data supporting the conclusion of this article will be made available by the authors, without undue reservation.
